# Evaluation of the In Vitro Antifungal Activity of Novel Arylsulfonamides against *Candida* spp.

**DOI:** 10.3390/microorganisms11061522

**Published:** 2023-06-08

**Authors:** Giovanna Ginestra, Teresa Gervasi, Francesca Mancuso, Federica Bucolo, Laura De Luca, Rosaria Gitto, Davide Barreca, Giuseppina Mandalari

**Affiliations:** 1 Department of Chemical, Biological, Pharmaceutical and Environmental Science, University of Messina, 98166 Messina, Italy; 2 Department of Biomedical and Dental Sciences and Morphofunctional Imaging, University of Messina, 98125 Messina, Italy

**Keywords:** *Candida albicans*, *Candida glabrata*, *Candida parapsilosis*, antifungal, arylsulfonamide, carbonic anhydrases

## Abstract

The antifungal activity of molecules belonging to the arylsulfonamide chemotype has previously been demonstrated. Here, we screened arylsulfonamide-type compounds against a range of *Candida* spp. and further established the structure–activity relationship based on a “hit compound”. A series of four sulfonamide-based compounds, N-(4-sulfamoylbenzyl) biphenyl-4-carboxamide (**3**), 2,2-diphenyl-N-(4-sulfamoylbenzyl) acetamide (**4**), N-(4-sulfamoylphenethyl) biphenyl-4-carboxamide (**5**) and 2,2-diphenyl-N-(4-sulfamoylphenethyl) acetamide (**6**), were tested against the American Type Culture Collection (ATCC) and clinical strains of *C. albicans*, *C. parapsilosis* and *C. glabrata*. Based on the fungistatic potential of prototype **3**, a further subset of compounds, structurally related to hit compound **3**, was synthesized and tested: two benzamides (**10**–**11**), the related amine 4-[[(4-4-((biphenyl-4-ylmethylamino)methyl) benzenesulfonamide (**13**) and the corresponding hydrochloride, **13^.^HCl**. Both amine **13** and its hydrochloride salt had fungicidal effects against *Candida glabrata* strain 33 (MFC of 1.000 mg/mL). An indifferent effect was detected in the association of the compounds with amphotericin B and fluconazole. The cytotoxicity of the active compounds was also evaluated. This data could be useful to develop novel therapeutics for topical use against fungal infections.

## 1. Introduction

Due to increased antifungal resistance, global effort has been focused on the identification of novel compounds from natural and synthetic sources [1,2]. *Candida albicans* is the most prevalent fungal pathogen in humans; in immunocompetent subjects, *C. albicans* is responsible for mucosal infections, including thrush and vaginitis, which could lead to invasive candidiasis in immunocompromised patients, especially with the increase in multidrug resistance. *Candida parapsilosis* also represents a significant cause of sepsis and infections, mostly in immunocompromised people and surgical patients. *Candida glabrata* is of special relevance in nosocomial infections due to its innate high resistance to antifungal agents, specifically azoles, and its ability to produce biofilm [3]. Biofilm formation is also common in *C. albicans* and *C. parapsilosis* and is especially relevant in bioprosthetic surfaces [4].

The most used antifungal compounds for treatment of *Candida* infections are amphotericin B (AMB) and fluconazole [5]. AMB is considered the gold standard in treatment of serious systemic mycoses in spite of its nephrotoxicity and adverse effects [6]. 

Fluconazole is widely used in clinical treatment of *Candida* infections, although it only has fungistatic activity against *C. albicans*. Therefore, in long-term treatment, *C. albicans* has the potential to acquire fluconazole resistance. A promising approach to increase fluconazole’s efficacy is identifying potential targets of drugs that can enhance its antifungal effect or even make it fungicidal [7].

We have recently demonstrated the in vitro potential of 1-(1H-indol-3-yl)-based compounds, endowed with antityrosinase activity, against *Candida* spp. [8], thus confirming that their enzyme inhibition continues to offer molecular targets in the identification of antifungal agents against *Candida* spp.

To further extend the chemical diversity of antifungal agents from synthetic sources, we moved our interest to a series of small molecules bearing a sulfonamide moiety as a zinc-binding group (ZBG) evaluated to reduce yeast virulence through β-carbonic anhydrase (β-CA) inhibition. Our idea was based on the consideration that *Candida* spp. employ β-CA to accomplish reversible hydration of CO_2_ as an essential process to guarantee survival. 

**Figure 1:**
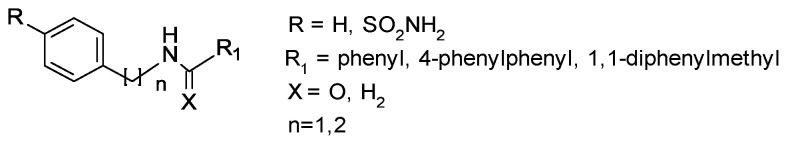
General chemical structure of studied compounds.

Earlier studies have well established that small molecules belonging to the sulfonamide class have proven to be antibacterial agents [9,10,11,12,13]; more recently, the antifungal activity of sulfonamides has been demonstrated [14,15,16]. Keeping this evidence in mind, in the present preliminary study, we screened arylsulfonamide-type compounds against a range of *Candida* spp. strains: *Candida albicans* ATCC 10531, *Candida parapsilosis* ATCC 22019, *Candida glabrata* DSM 70614, three clinical isolates of *Candida albicans*, two clinical isolates of *Candida glabrata* and two clinical isolates of *Candida parapsilosis*. We herein evaluate the activity of our disclosed and unpublished compounds related to arylsulfonamide chemotypes, for which the general chemical structure is reported in Figure 1. The abovementioned compounds were selected with the aim to explore the impact of several structure modifications on antifungal effects. In detail, we analyzed the roles of distinct structural determinants as follows: (a) the role of the sulfonamide function (R substituent); (b) the variation of the pKa value that is related to the presence of amine (X=H_2_) or amide (X=O) functionality and (c) improvement of lipophilic properties by insertion of R_1_ and R_2_ aromatic tails.

## 2. Materials and Methods

### 2.1. General Chemistry

Merk Life Science S.r.l. (Milan, Italy) and Thermo Fisher Scientific - Alfa Aesar (Erlangen, Germany) provided all chemicals, reagents and solvents of reagent grade, which were used without further purification. Melting points were determined on a Buchi B-545 apparatus (BUCHI Labortechnik AG Flawil, Flawil, Switzerland) and uncorrected. Combustion analysis (C, H, N) was carried out using a Carlo Erba Model 1106 Elemental Analyzer; we established the purity of all compounds, and the results confirmed ≥95% purity. Reaction progress was monitored with thin-layer chromatography (TLC) using glass-backed TLC 60 G silica gel plates (0.25 mm) containing the fluorescence indicator F254. Chromatograms were visualized with UV light (λ = 254 nm/366 nm) or by staining with ninhydrin solution. ^1^H NMR and ^13^C NMR spectra were measured in dimethylsulfoxide-d_6_ (DMSO-d_6_) with a Varian Gemini 500 spectrometer (Varian Inc. Palo Alto, CA, USA). Chemical shifts are reported in δ (ppm) and coupling constants (*J*) in hertz. All exchangeable protons were confirmed with addition of D_2_O. NMR spectra for compounds **10**, **11** and **13 HCl** are reported in the Supplementary Materials.

### 2.2. General Synthetic Procedure for Amide Derivatives 3–6, 10–11

The four sulfonamides, **3**–**6**, were obtained by following a previously reported procedure [17,18]; their physicochemical properties and spectroscopic data were in good agreement with previous data [17,18]. *N*-benzylbenzamide (**10**) and *N*-benzylbiphenyl-4-carboxamide (**11**) have already been reported in the literature by other authors [19,20]. To obtain compounds **10**–**11**, we employed a different synthetic procedure, as detailed below; we performed structural determinations with spectroscopic measurements, and the obtained data were coherent with those reported in the literature [19,20].

In detail, to a solution of suitable amine derivative (1.25 mmol) in DCM/DMF (6 mL, 2:1, *v*/*v*), a mixture of N,N-diisopropylethylamine (DIPEA, 2.5 mmol) and the aroyl/alkyl chloride derivative (1 mmol) was added. The reaction mixture was left to be stirred at room temperature for an appropriate time by monitoring the reaction completion with thin-layer chromatography (MeOH:DCM, 90:10). Then, the solvent was removed under vacuum and the resulting residue dissolved in H_2_O (10 mL). The resulting residue was dissolved in H_2_O (10 mL) and extracted with EtOAc (3 × 10 mL). The combined organic layers were dried over Na_2_SO_4_ and concentrated until dry under reduced pressure. The crude product was crystallized with Et_2_O and EtOH to provide the desired derivatives, **3**–**6** and **10**–**11**, as powder.

*N-benzylbenzamide (* **10** *)*

Yield: 56%; m.p.105–107 °C. ^1^H NMR (500 MHz, DMSO-d_6_): δ ppm 4.49 (m, 2H, CH_2_), 7.22 (m, 1H, ArH), 7.26 (m, 4H, ArH), 7.49 (m, 3H, ArH), 7.89 (m, 2H, ArH), 9.02 (br s, 1H, NH). Anal. For C_14_H_13_NO: C, 79.59%; H, 6.20%; N, 6.63%. Found: C, 79.62%; H, 6.21%; N, 6.73%.

*N-benzylbiphenyl-4-carboxamide (* **11** *)*

Yield: 81%; m.p. 90–91 °C. ^1^H NMR (500 MHz, DMSO-d_6_): δ ppm 4.50 (m, 2H, CH2), 7.21 (m, 1H, ArH), 7.27 (m, 4H, ArH), 7.38 (m, 1H, ArH), 7.49 (m, 2H, ArH), 7,70–7.79 (m, 4H, ArH), 7.99 (m, 2H, ArH), 9.09 (br s, 1H, NH). Anal. For C_20_H_17_NO: C, 83.59%; H, 5.96%; N, 4.87%. Found: C, 83.51%; H, 5.86%; N, 4.99%. 

### 2.3. Synthesis of 4-[[(4-4-((biphenyl-4-ylmethylamino)methyl)benzenesulfonamide (***13*** and Its Hydrochloride ***13^.^HCl***)

To a solution of 4-(aminomethyl)benzenesulfonamide (250 mg, 1.2 mmol) in DMF (3 mL), K_2_CO_3_ (155 mg, 1.12 mmol) and 4-phenylbenzyl chloride (227 mg, 1.2 mmol) were added. The reaction was stirred at 100 °C under reflux for 24 h. The reaction was quenched with a saturated NaHCO_3_ aqueous solution, and then the residue was dissolved in H_2_O (10 mL) and extracted with EtOAc (3 × 10 mL); the organic layers were dried over Na_2_SO_4_ and concentrated until dry under reduced pressure to give a crude product that was crystallized with Et2O and EtOH to afford compound **13** as white powder. The hydrochloride of derivative **13** was obtained by treating the abovementioned organic layer with HCl 37% drops, so the amine salt was collected with vacuum filtration, dried and recrystallized with Et_2_O and EtOH to obtain the desired **13^.^HCl**.

*4-[[(4-4-((Biphenyl-4-ylmethylamino)methyl)benzenesulfonamide (* **13** *)*

Yield: 88%; m.p.156–158 °C. ^1^H NMR (500 MHz, DMSO-d_6_):δ ppm 2.78 (bs, 1H, NH), 3.72 (s, 2H, CH_2_), 3.77 (s, 2H, CH_2_), 7.30 (br s, 2H, NH_2_), 7.33–7.36 (m, 1H, ArH), 7.43–7.46 (m, 4H, ArH), 7.55 (d, J= 7.5 Hz, 2H, ArH), 7.62 (d, J = 7.5 Hz, 2H, ArH), 7.65 (d, J = 7.7 Hz, 2H, ArH), 7.78 (d, J = 7.7 Hz, 2H, ArH). Anal. For C_20_H_20_N_2_O_2_S: C, 68.16%; H, 5.72%; N, 7.95%, Found: C, 68.18%; H, 5.70%; N, 7.97%.

*4-[[(4-4-((Biphenyl-4-ylmethylamino)methyl)benzenesulfonamidehydrocholoride (* **13^.^HCl** *)*

Yield: 62%; m.p. > 400 °C (dec); ^1^H NMR (500 MHz, DMSO-d_6_): δ ppm 4.19–4.31 (m, 4H, 2 CH_2_), 7.36–7.53 (m, 5H, ArH and SO_2_NH_2_), 7.62–7.80 (m, 8H, ArH), 7.83–7.92 (m, 2H, ArH), 9.80 (br s, 2H, NH.HCl).^13^C-NMR (126 MHz, DMSO-d6): 49.2, 49.6, 125.6, 125.7, 125.8, 126.6, 126.8, 126.9, 128.9, 129.1, 130.6, 130.8, 130.9, 135.7, 139.4, 140.7, 144.5; Anal. For C_20_H_21_ClN_2_O_2_S: C, 61.77%; H, 5.44%; N, 7.20%. Found: C, 61.79%; H, 5.42%; N, 7.25%.

### 2.4. Microbial Strains and Culture Conditions

The following strains were used: *C. albicans* ATCC 10231, 3 clinical strains of *C. albicans* (12, 13, 16), *C. parapsilosis* ATCC 22019, 2 clinical strains of *C. parapsilosis* (30, 34), *C. glabrata* DSM 70614 and 2 clinical strains of *C. glabrata* (9, 33). Strains were grown in Sabouraud Dextrose Broth and Agar (Oxoid) and assays performed in RPMI 1640 (Sigma, Italy) at 30 °C for 24 h, as previously reported [8]. For minimal fungicidal determination and killing curves, Sabouraud Dextrose Agar was used.

### 2.5. Susceptibility Studies

All derivatives were dissolved in DMSO at a concentration of 10 mg/mL. The minimum inhibitory concentration (MIC) and the minimum fungicidal concentration (MFC) were determined following the CLSI guidelines at concentrations ranging between 2.0 and 0.0039 mg/mL [21]. A positive control using fluconazole was included in each assay, whereas acetazolamide was included as a β-carbonic anhydrase (β-CA) inhibitor. The MIC was defined as the lowest concentration of compounds that inhibited the visible growth of the tested strains after incubation. The MFC was defined as the lowest compound concentration that killed 99.9% of the final inocula after 24–48 h incubation. The MFC was determined by transferring an aliquot (20 μL) from a clear sample onto an agar plate, which was incubated at 30 °C for 48 h.

To test the efficacy of the combination of compounds **3**, **13** and **13^.^HCl** and the antifungal compounds fluconazole and AMB (Sigma Aldrich, Italy), the “checkerboard” procedure was performed [22].

The MIC data for the compound 3 derivatives and each antifungal compound were used to calculate the fractional inhibitory concentrations (FICs).

For two antibacterial agents, A and B, acting individually or in combination:

The FIC index was calculated as follows: FIC index = FIC A + FIC B, where FIC A is the MIC of drug A in the combination/the MIC of drug A alone and FIC B is the MIC of drug B in the combination/the MIC of drug B alone.

All experiments were performed in triplicate on three independent days.

### 2.6. Evaluation of Cytotoxicity Assay

Human mononuclear cells (HMNCs) were bought from Sigma-Aldrich and processed following the details supplied by the seller. During the cytotoxicity assay, cells (1 × 10^5^/mL) were incubated in a complete medium (Blood Cell Growth Medium supplied by Merk) without or with 0.061, 0.125, 0.250, 0.500, 0.750 or 1.000 mg/mL of compounds **3**, **13** and **13-HCl** for 24 h at 37 °C. An aliquot of the cells was adequately diluted and mixed with trypan blue 0.4% (1:1 *v*:*v*) and the cells counted using a hemocytometer. Results were expressed as the viable percentage (ratio of unstained cells to the total number of cells). Another aliquot of cells was centrifuged at 2500 rpm for 5 min and the supernatant analyzed to detect the release of lactate dehydrogenase. The supernatant was also utilized to check cytotoxicity by measuring lactate dehydrogenase (LDH) release from damaged cells into the culture medium. LDH activity in the medium was determined using a commercially available kit from BioSystems S.A. The tested compounds, at the concentrations utilized in these experiments, did not interfere with LDH determination. Control samples and samples treated with the solvent only used to dissolve the tested compounds (V) were also included in each test. The LDH activity in the medium was expressed as a function of the total amount of the enzyme present in the control (cells without tested compounds) released after induced total cell lysis by sonication. The pocked cells were resuspended and washed three times in a phosphate saline buffer, homogenized with a sonicator and utilized for caspase 3 activation following a procedure described in the literature [23,24].

### 2.7. Statistical Analysis

Data are expressed as mean ± standard deviation (S.D.). Statistical analyses were performed with one-way analysis of variance (ANOVA). The significance of the difference from the respective controls for each experimental test condition was assayed using Tukey’s test for each paired experiment. *p* < 0.05 was considered statistically significant.

## 3. Results

### 3.1. Chemistry

**Scheme 1:**
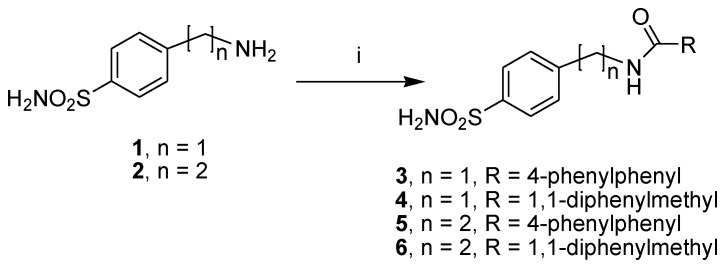
Synthetic scheme of sulfonamide-based compounds **3**–**6**. Reagents and conditions: RCOCl, DIPEA, DCM/DMF (2:1, *v*/*v*), rt, 2–4 h.

The first subset of four sulfonamide-based compounds, N-(4-sulfamoylbenzyl)biphenyl-4-carboxamide (**3**), 2,2-diphenyl-N-(4-sulfamoylbenzyl)acetamide (**4**), N-(4-sulfamoylphenethyl)biphenyl-4-carboxamide (**5**) and 2,2-diphenyl-N-(4-sulfamoylphenethyl) acetamide (**6**), was retrieved in our library; therefore, these compounds were re-synthesized [17,18] by employing the synthetic procedure reported in Scheme 1, with slight modifications. In detail, these compounds were prepared with the coupling reaction of the 4-(aminomethyl) benzenesulfonamide hydrochloride (**1**) or 4-(aminoethyl) benzenesulfonamide (**2**) and the suitable aroyl chlorides in basic conditions.

**Scheme 2:**
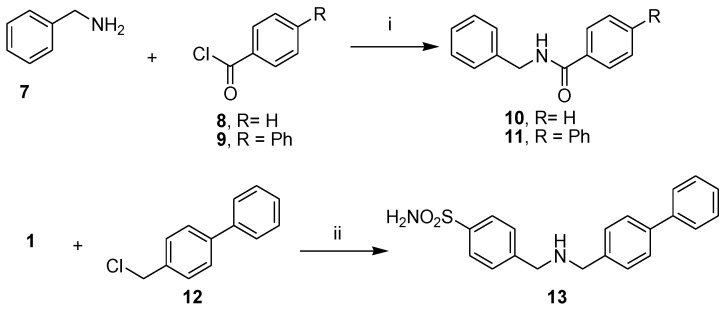

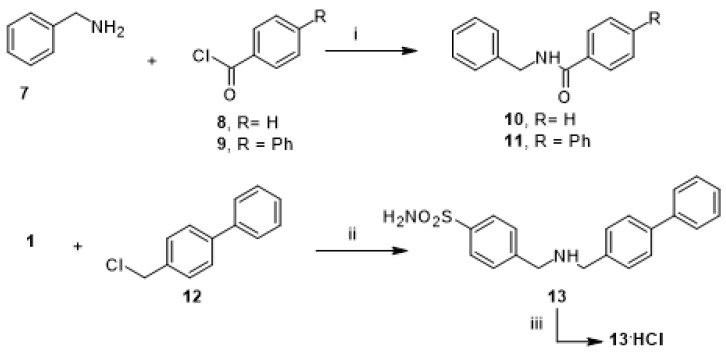
Synthetic scheme of amides **10**–**11** and amine **13**. Reagents and conditions: (i) DIPEA, DCM/DMF (2:1, *v*/*v*), rt, 1–2 h; (ii) K_2_CO_3_, DMF, 100 °C, 24 h; (iii) HCl 37% drops, rt.

To introduce some modification to promising hit compound **3**, we prepared a further subset of analogue compounds, **10**, **11**, and **13**, by following the synthetic route described in Scheme 2. The desired N-benzylbenzamide (**10**) was obtained with the reaction of benzoyl chloride (**8**) to benzylamine (**7**), which was used as a starting material to obtain N-benzylbiphenyl-4-carboxamide (**11**) by coupling with 4-phenylbenzoyl chloride (**9**). Finally, the N-alkylation of 4-(aminomethyl) benzenesulfonamide (**1**) with 4-phenylbenzyl chloride (**12**) gave the free amine 4-[[(4-4-((biphenyl-4-ylmethylamino)methyl) benzenesulfonamide (**13**), which provided the corresponding hydrochloride (**13^.^HCl**) by treatment with HCl aqueous solution.

### 3.2. Antifungal Effects

**Figure 2:**
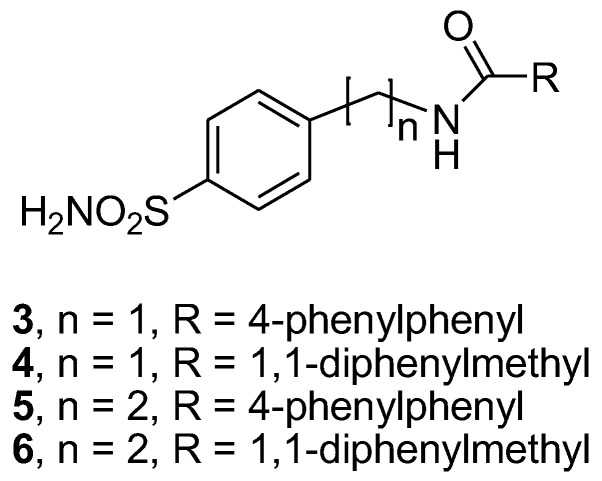
Chemical structure of studied compounds **3**–**6**.

The MIC values for the first series of our previously synthesized sulfonamide-based compounds **3**–**6** (Figure 2) are reported in Table 1. All negative controls that used DMSO as a solvent indicated the complete absence of inhibition of all the strains tested (data not shown). The MIC and MFC values of fluconazole were obtained as previously reported [25]. The results showed a fungistatic activity of compound **3** against all tested strains at concentrations ranging between 0.125 and 1 mg/mL, with the exceptions of *Candida glabrata* DSM 70614 and one clinical strain of *Candida glabrata* (strain 9).

**Figure 3:**
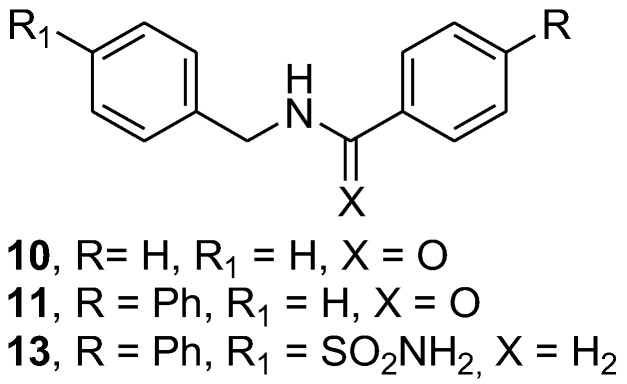
Chemical structure of studied compounds **10**, **11** and **13**.

With these promising data in hand, “hit structure” **3** was modified to expand our series of compounds and to collect structure–activity relationship information on the antifungal activity for this series of compounds (Table 2). In detail, the structure–function relationship was explored through the synthesis of a novel series of a few analogues (Figure 3) bearing a different aromatic tail as well as through the variation in the linking group. The best outcome resulted by means of the carbonyl (C=O) to methylene (CH_2_) exchange that furnished the corresponding amine, **13**; this compound proved to be effective against *Candida albicans* strain 16 and *Candida glabrata* strain 33. Moreover, an effect against *Candida glabrata* strain 33 was detected at a concentration of 1.000 mg/mL. Interestingly, the corresponding hydrochloride salt, **13^.^HCl**, was active on several strains, showing inhibitory effects against *Candida glabrata* strain 33 (MIC of 0.250 mg/mL) and *Candida albicans* strain 12 and strain 16 (MIC values ranging between 0.500 and 1.000 mg/mL). A fungicidal effect of compound **13** was detected against *Candida glabrata* strain 33 (MFC of 1.000 mg/mL).

Table 3 reports the FIC indices calculated for the three most effective compounds (**3**, **13**, **13^.^HCl**) and each antifungal compound against *C. albicans* strain 12, *C. albicans* strain 16 and *C. glabrata* strain 33. To study the effect of the combinations of the active substances, we selected the most sensitive strains to the tested compounds.

Given that FIC index interpretation depends on which definition is used, here, we have reported the value as synergistic if the FIC index is ≤0.5, additive or indifferent if it is >0.5 but ≤4 and antagonistic if it is >4 [26].

All the combinations showed an indifferent effect, whereas no antagonistic interaction was observed against any tested strain.

### 3.3. Cytotoxicity Assay

**Figure 4:**
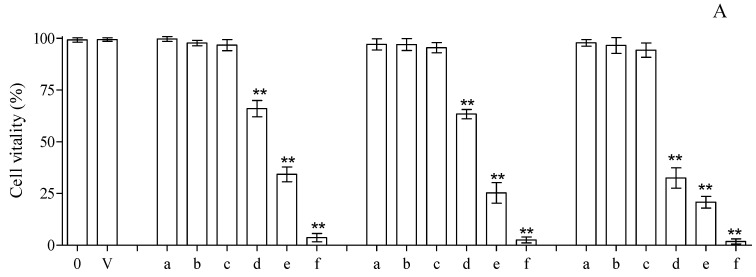

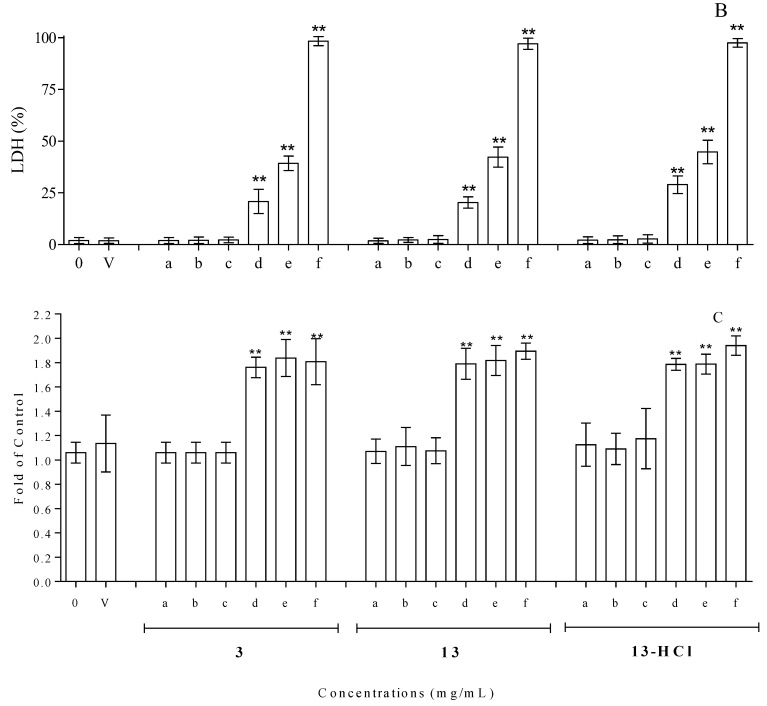
Cytotoxicity evaluation, utilizing HMNCs, of compounds **3**, **13** and **13^.^HCl** at different concentrations after 24 h incubations and at 37 °C. Cell vitality and integrity were analyzed with trypan blue staining (**A**) and LDH release (**B**); apoptosis was analyzed with caspase 3 activation (**C**). The tested concentrations were 0.0 (0), 0.062 (a), 0.125 (b), 0.250 (c), 0.500 (d), 0.750 (e) and 1.000 (f) mg/mL. A sample treated with only the solvent was utilized to dissolve the tested compounds (V). HMNCs incubated with only the solvent were utilized to dissolve the tested compounds. The asterisks (**) indicate significant differences (*p* < 0.05) in the samples versus the control (0.000 mg/mL).

The cytotoxicity assay, carried out by means of trypan blue coloration and treating isolated lymphocytes with different amounts of compounds **3**, **13** and **13^.^HCl** for 24 h at 37 °C, based on the MIC values (0.065–1.000 mg/mL), revealed good tolerability vs. the control at concentrations of up to 0.250 mg/mL for all the tested compounds. On the contrary, a net increase in cell mortality was found at higher concentrations, reaching mortalities of ~35, 66 and 97% for cells incubated with compound **3** at concentrations of 0.500, 0.750 and 1.000 mg/mL, respectively (Figure 4A). The results of the trypan blue exclusion assay were in line with the data obtained when monitoring the LDH (commonly utilized as a marker of membrane integrity) in the same compounds. Concentrations of up to 0.250 mg/mL showed values of LDH release superimposable with that obtained with the control, while superior concentrations resulted in clear increases in LDH release (Figure 4B). Finally, the cytotoxicity evaluation was carried out by monitoring caspase 3 activation in HMNCs to evaluate the possible activation of apoptotic events. As shown in Figure 4C, the results obtained with the activation of caspase 3 resulted in statistically significant changes, with respect to the untreated samples, only in the concentration range of 0.500–1.000 mg/mL, while no activation of apoptotic events was detected at lower concentrations.

## 4. Discussion

Based on the experimental evidence that arylsulfonamide-based CA inhibitors have been successfully employed to discover antimicrobial agents, in the present work, we have disclosed a few compounds with fungistatic and fungicidal activity against clinical isolates of *Candida albicans* and *Candida glabrata*, respectively, and could potentially be used for topical formulations, either alone or in combination with available antifungal drugs. Our study began with a preliminary screening of our chemical library; we found the “hit structure” N-(4-sulfamoylbenzyl) biphenyl-4-carboxamide, **3**, which was further modified in order to better understand the structure–function relationship that controls antifungal activity. The corresponding amine derivative, **13**, and its hydrochloride salt acquired fungicidal potential against *Candida glabrata* strain 33, though they were able to inhibit fewer strains. Compound **13^.^HCl** was slightly more effective than the free amine, which might be rationalized by the improved water solubility of hydrochloride compared with the free amine [27].

On the other hand, standard and clinical *Candida parapsilosis* strains were sensitive to **3** only, thus indicating that the presence of amide functionality is relevant for optimizing activity against all tested strains, with the exceptions of *Candida glabrata* strain 9 and *Candida glabrata* DSM 70614. When the sulfonamide group was removed from lead compound **3**, the antifungal activity was lost, as was found for compound **11**. Likewise, the structural modification made to compound **10** led to activity loss. We can hypothesize that the sulfonamide moiety might have contributed to the effect on β-CA in the *Candida* spp., even if the presence of sulfonamide was not sufficient to exert antifungal activity, as found for our tested sulfonamide-based compounds **4**–**6**.

It has been established that the well-known CA inhibitor acetazolamide determines reductions in blood pH given the increased carbonic acid, which has a reversible reaction into bicarbonates and protons [28,29].

Clinically used sulfonamides and sulfamates, including acetazolamide, ethoxzolamide, methazolamide, dorzolamide, topiramate and celecoxib, generally proved to be effective against the CA of *Saccharomyces cerevisiae*, with KIs in the range of 82.6–133 nM [30]. *C. glabrata* encoded for β- CA, indicated with the acronym CgNce103 or CgCA, with a kcat of 3.8 × 10^5^s^−1^ and a kcat/KM of 4.8 × 10^7^ M^−1^s^−1^ [31]. Acetazolamide, ureido-benzene-sulfonamides, sulfamates and sulfamides effectively inhibited CgNce103, with KIs in the range of 4.1–115 nM; poly(amidoamine) dendrimers that incorporated benzenesulfonamide were also able to inhibit CgNce103 [32]. Akdemir et al. (2016) also demonstrated the effects of isatin-containing sulfonamides on CaNce103 from *C. albicans* and CgNce103 from *C. glabrata* [33]. Due to the significant resistance of *C. glabrata* to several antifungal agents, sulfonamide inhibition of CgNce103 may provide alternative means to prevent fungal growth [14]. In the present work, we have demonstrated the fungicidal potential of two sulfonamide-based compounds on *C. glabrata* strain 33, whereas acetazolamide did not have any effect (MIC > 2 mg/mL).

It is well-accepted that CA inhibition may impair growth of microorganisms [34]. In the yeasts *Candida albicans* and *Malassezia globosa*, the expression of β-CA was essential for the growth of the strains. However, it is possible that our active agent, **3**, and its active derivatives, **13** and **13^.^HCl**, possess suitable structural requirements, addressing a higher specificity for the CA expressed by the tested strains compared with acetazolamide. This could explain the lack of activity toward fungal growth by acetazolamide. It is possible to suppose that the antifungal activity of **3**, **13** and **13^.^HCl** might be related to a synergistic effect toward different molecular targets, which could affect yeast growth.

Combinations of antimicrobial agents can have different effects, including indifferent, additive, synergistic and antagonistic. The indifferent effect is observed when a blend of antimicrobial agents or a combination of an antibacterial agent and an inactive substance has an identical effect to that of the most active constituent.

Additive effects occur when a mixture of antibacterial agents has an effect equal to the sum of the effects of all components. The synergistic effect is observed when a combination of antibacterial agents has a greater effect than the added effects of each constituent. Antagonistic effects of a treatment are shown when reduced activity is observed relative to the effect of the most efficient individual constituent.

These effects can be quantified by application of mathematical expressions such as the fractional inhibitory concentration (FIC) [35].

The association between compounds **3**, **13** and **13^.^HCl**, with both fluconazole and amphotericin B, mostly resulted in an additive effect against three tested strains. Indifference in tending to synergism was observed between compound **13^.^HCl** and fluconazole against *C. albicans* 16, indicating the most promising combination.

Choi et al. [36] have recently optimized a series of antifungal agents by substituting various aryl ring, alkyl chain and methyl groups; they revealed that the presence of a suitable lipophilic diphenyl tail significantly improves antimicrobial activity, thus confirming an effect on fungal cell wall integrity by activation of several cell wall integrity pathways. In the future, we will aim to evaluate the potential of our series of sulfonamide-based compounds on the cell wall integrity of our *Candida* spp.

## 5. Conclusions

In the present work, we discovered the fungistatic and fungicidal effects of existing and novel arylsulfonamide-type compounds against ATCC and clinical strains of *Candida* spp. The cytotoxicities of the compounds were also examined. Overall, our preliminary studies confirmed that arylsulfonamide derivatives bearing specific structural requirements might be explored as antifungal agents towards *Candida* spp., determining impairment of fungal growth by means of innovative targets. These data could be relevant to develop novel topical formulations against fungal infections. Further research could focus on generating novel arylsulfonamide derivatives to be tested against a broader range of drug-resistant fungi, alone or in combination with available antifungal drugs.

Moreover, we could hypothesize that CAs might be involved in antifungal effects; however, additional studies are necessary to confirm the role of CAs inhibition in providing biological efficacy. Further studies are also warranted to elucidate the potential applications of these compounds, specifically in relation to their mechanisms of action within host cells.

## Figures and Tables

**Table 1:** MICs of arylsulfonamides **3**–**6** (expressed as mg/mL) against *Candida* spp. Results represent the means of triplicate independent assays.

Strain	3	4	5	6
*Candida glabrata* Strain *9*	>1.000	>1.000	>1.000	>1.000
*Candida glabrata* Strain *33*	0.125–0.250	>1.000	>1.000	>1.000
*Candida glabrata* DSM 70614	>1.000	>1.000	>1.000	>1.000
*Candida parapsilosis* Strain *30*	0.250	>1.000	>1.000	>1.000
*Candida parapsilosis* Strain *34*	0.500	>1.000	>1.000	>1.000
*Candida parapsilosis* ATCC 22019	0.125–0.250	>1.000	>1.000	>1.000
*Candida albicans* Strain *12*	0.250	>1.000	>1.000	>1.000
*Candida albicans* Strain *13*	0.250	>1.000	>1.000	>1.000
*Candida albicans* Strain *16*	0.125–0.250	>1.000	>1.000	>1.000
*Candida albicans* ATCC 10231	0.250	>1.000	>1.000	>1.000

**Table 2:** MICs of compounds **10**, **11**, **13** and **13^.^HCl** related to “hit compound” **3** (expressed as mg/mL) against *Candida* spp. Results represent the means of triplicate independent assays.

Strain	3	10	11	13	13^.^HCl
*Candida glabrata* Strain 9	>1.000	>1.000	>1.000	>1.000	>1.000
*Candida glabrata* Strain 33	0.125–0.250	>1.000	>1.000	0.500	0.250
*Candida glabrata* DSM 70614	>1.000	>1.000	>1.000	>1.000	>1.000
*Candida parapsilosis* Strain 30	0.250	>1.000	>1.000	>1.000	>1.000
*Candida parapsilosis* Strain 34	0.500	>1.000	>1.000	>1.000	>1.000
*Candida parapsilosis* ATCC 22019	0.125–0.250	>1.000	>1.000	>1.000	>1.000
*Candida albicans* Strain 12	0.250	>1.000	>1.000	>1.000	0.500
*Candida albicans* Strain 13	0.250	>1.000	>1.000	>1.000	>1.000
*Candida albicans* Strain 16	0.125–0.250	>1.000	>1.000	1.000	0.500–1.000
*Candida albicans* ATCC 10231	0.250	>1.000	>1.000	>1.000	>1.000

**Table 3:** FIC indices of the associations between compounds **3**, **13** and **13^.^HCl** with the reference antifungal compounds (fluconazole and amphotericin B) against *C. albicans* 12, *C. albicans* 16 and *C. glabrata* 33.

Mixture	*C. glabrata 33*	*C. albicans 16*	*C. albicans 12*
Compound **3**/Fluconazole	2.00 (FIC A = 1.00, FIC B = 1.00)	1.01 (FIC A = 0.0156, FIC B = 1.00)	2.01 (FIC A = 0.0156, FIC B = 2.00)
Compound **13**/Fluconazole	1.01 (FIC A = 1.00, FIC B = 0.0078)	1.12 (FIC A = 0.12, FIC B = 1.00)	0.99 (FIC A = 0.49, FIC B = 0.50)
Compound **13**^.^**HCl**/Fluconazole	2.00 (FIC A = 1.00, FIC B = 1.00)	0.75 (FIC A = 0.49, FIC B = 0.26)	1.03 (FIC A = 0.0312, FIC B = 1.00)
Compound **3**/Amphotericin B	1.01 (FIC A = 1.00, FIC B = 1.0076)	1.01 (FIC A = 0.0078, FIC B = 1.00)	1.01 (FIC A = 0.0078, FIC B = 1.00)
Compound **13**/Amphotericin B	1.02 (FIC A = 1.00, FIC B = 0.0156)	2.24 (FIC A = 2.00, FIC B = 0.24)	1.50 (FIC A = 1.00, FIC B = 0.50)
Compound **13**^.^**HCl**/Amphotericin B	1.01 (FIC A = 1.00, FIC B = 0.0076)	1.01 (FIC A = 0.0156, FIC B = 1.00)	1.01 (FIC A = 0.0156, FIC B = 1.00)

## Data Availability

Data are available upon request from the corresponding authors.

## Supplementary Materials

The following supporting information can be downloaded at: https://www.mdpi.com/article/10.3390/microorganisms11061522/s1, Figures S1–S4: NMR spectra of new synthesized compounds.
