# Supplementary material for: Evaluation of the In Vitro Antifungal Activity of Novel Arylsulfonamides against Candida spp
Source: Microorganisms. 2023 Jun 8;11(6):1522. doi: 10.3390/microorganisms11061522 (PMC10304118; doi:10.3390/microorganisms11061522)
Supplement: Supplementary file 1 [file microorganisms-11-01522-s001.zip › microorganisms-2282175-supplementary.pdf]

Supplementary Material

Evaluation of the *in vitro* antifungal activity of novel arylsulfonamides against *Candida* spp.

Giovanna Ginestra[1], Teresa Gervasi[2], Francesca Mancuso[1], Federica Bucolo[1], Laura De Luca[1], Rosaria Gitto[1], Davide Barreca[1,]* and Giuseppina Mandalari[1,]*

[1] Department of Chemical, Biological, Pharmaceutical and Environmental Science, University of Messina, Messina, Italy

[2] Department of Biomedical and Dental Sciences and Morphofunctional Imaging, University of Messina, Messina, Italy

* Correspondence: gmandalari@unime.it; dbarreca@unime.it; Tel.: 0030 090 6766593

Contents

Figures S1-S4 NMR spectra of new synthesized compounds 10, 11, 13·HCl

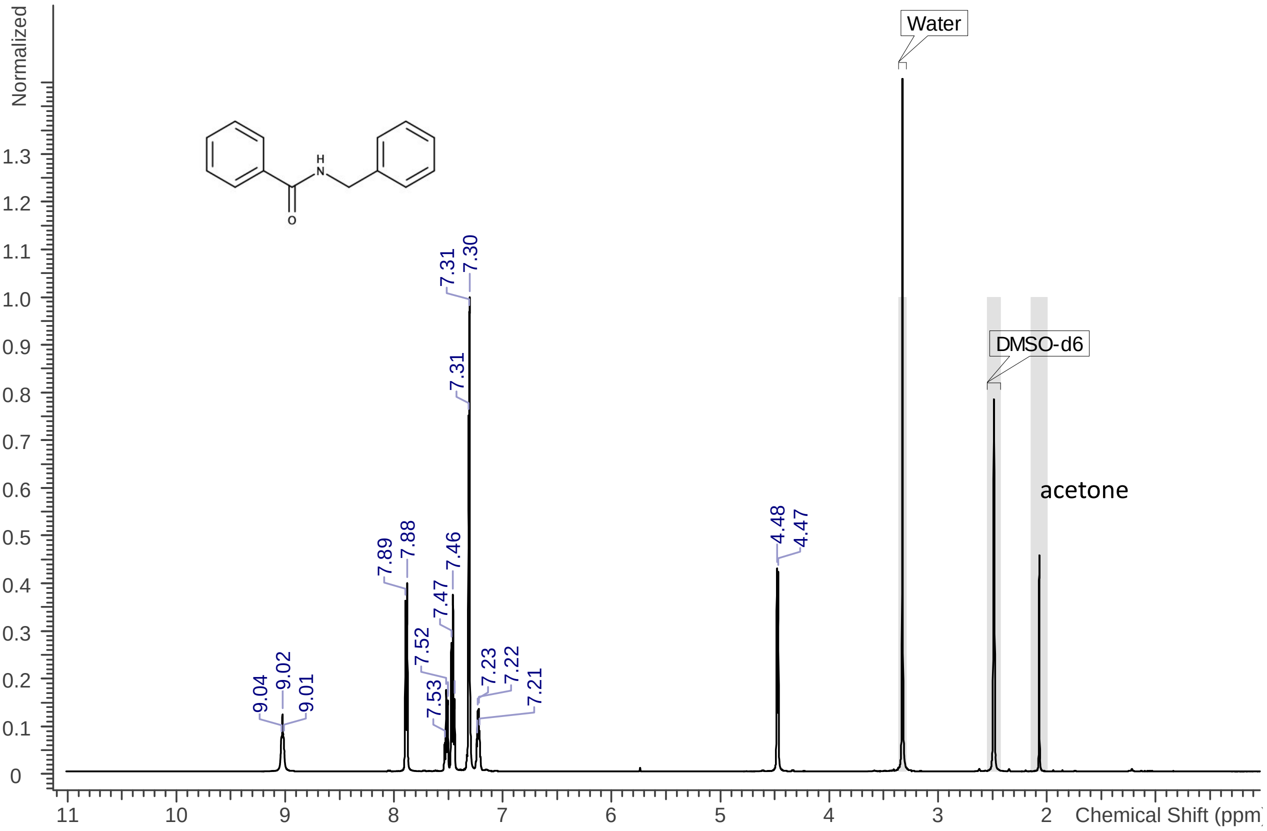

Figure S1 ^{1}H NMR spectrum of *N*-benzylbenzamide **(10)** (500 MHz, DMSO-*d*$_6$)

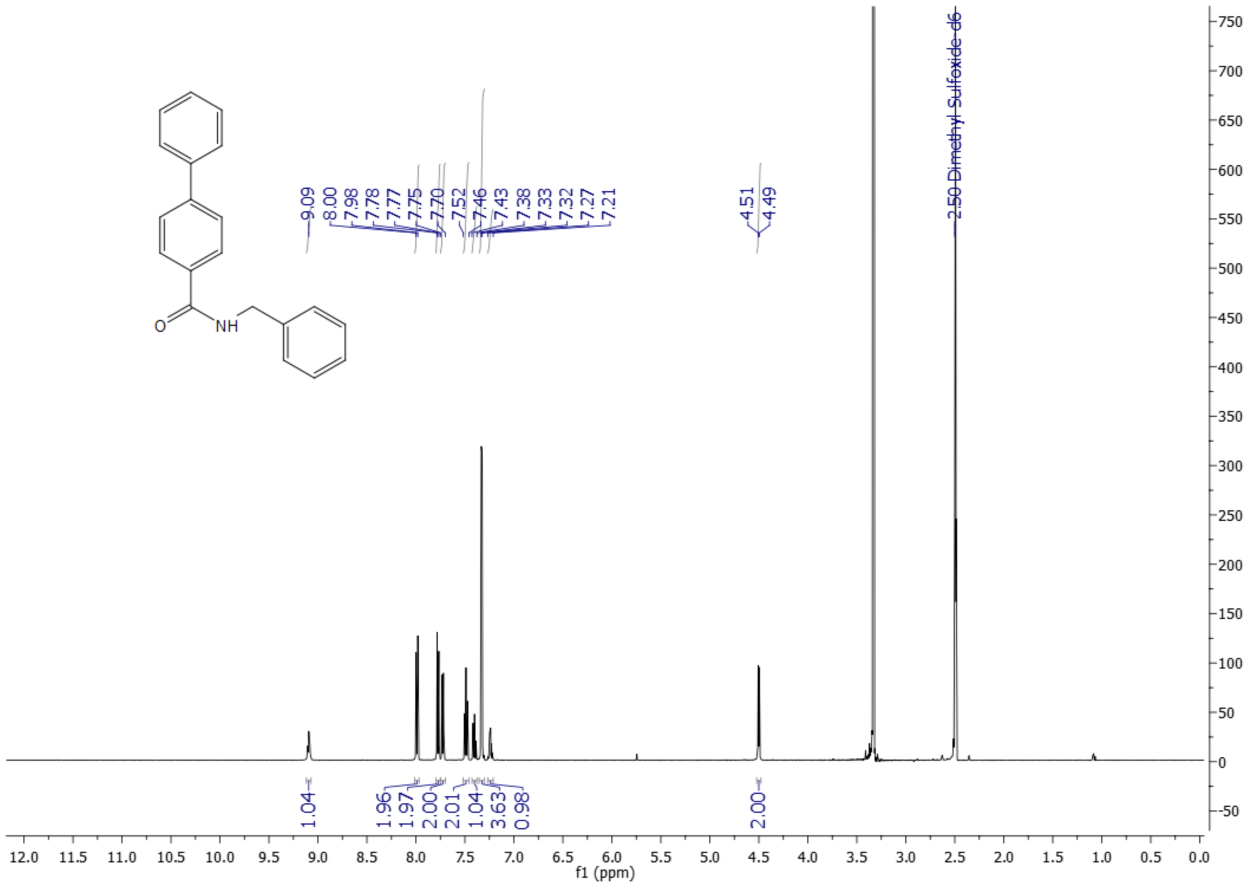

Figure S2 ^{1}H NMR spectrum of *N*-benzylbiphenyl-4-carboxamide (**11**) (500 MHz, DMSO-d_6)

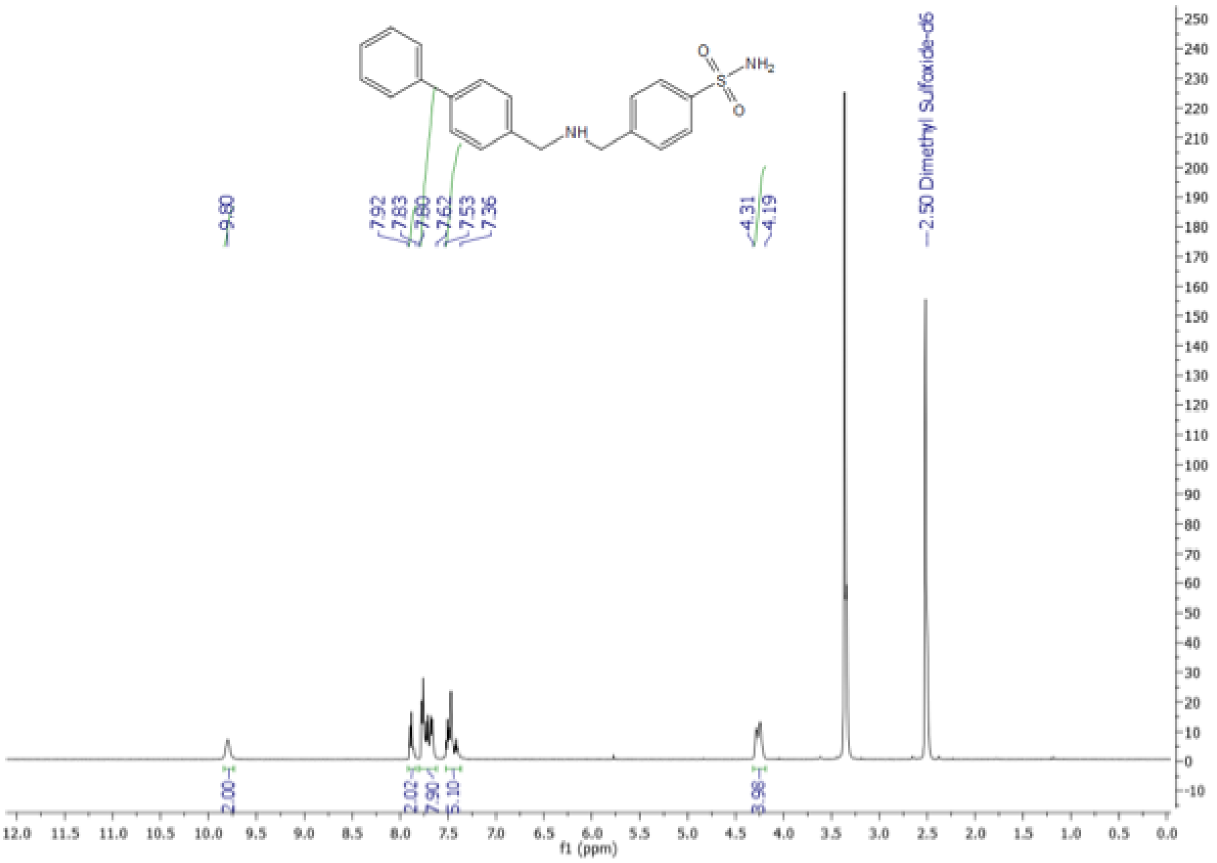

Figure S3 ^{1}H NMR spectrum of 4-{[({[1,1′-biphenyl]-4-yl}methyl)amino]methyl}benzene-1-sulfonamide hydrocholoride (**13·HCl**) (500 MHz, DMSO-*d*$_6$)

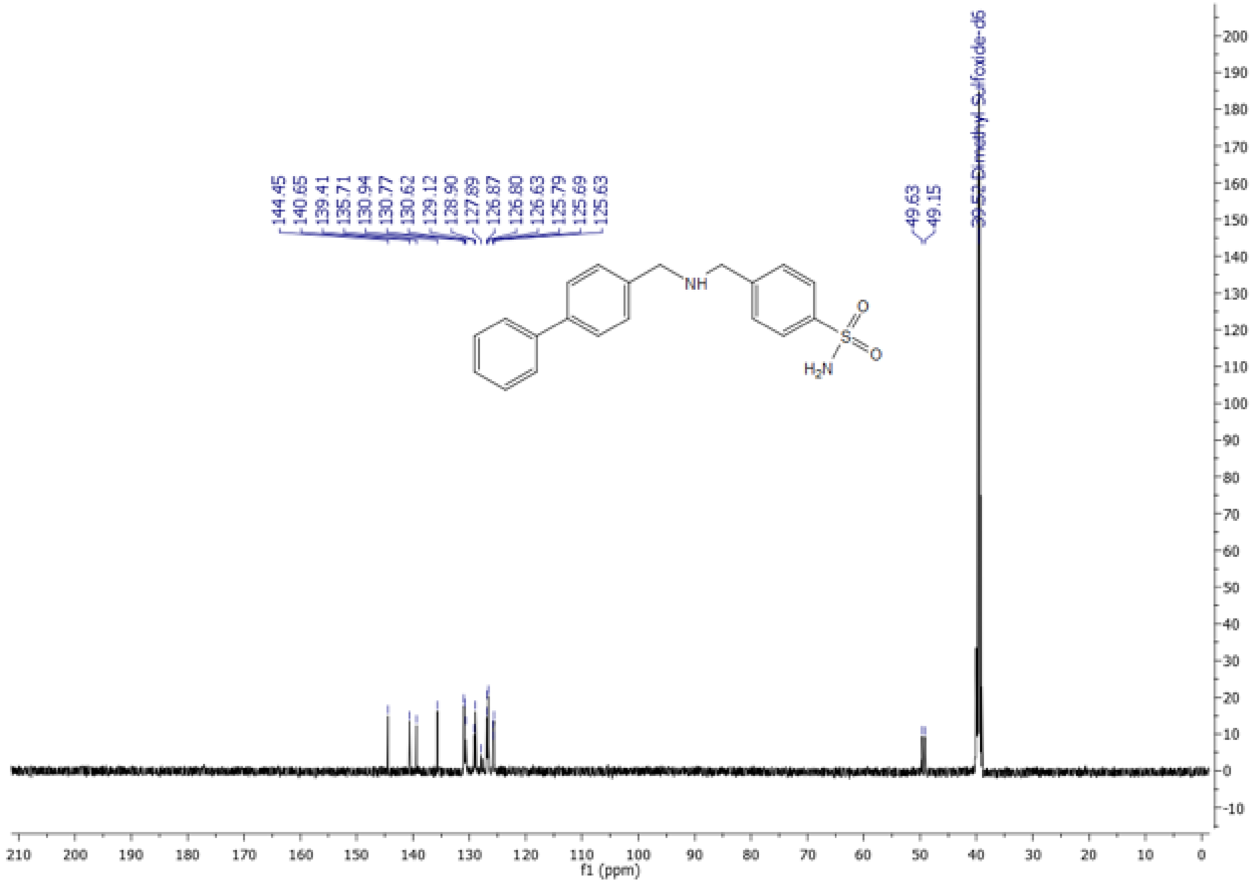

Figure S4 ^{13}C NMR spectrum of 4-{[({[1,1′-biphenyl]-4-yl}methyl)amino]methyl}benzene-1-sulfonamide hydrocholoride (**13·HCl**) (126 MHz, DMSO-d_6)
